# Evolutionary trends in the family Curimatidae (Characiformes): inferences from chromosome banding

**DOI:** 10.3897/CompCytogen.v10i1.6316

**Published:** 2016-01-22

**Authors:** Tatiane Ramos Sampaio, Larissa Bettin Pires, Natália Bortolazzi Venturelli, Mariana Campaner Usso, Renata da Rosa, Ana Lúcia Dias

**Affiliations:** 1 Departamento de Biologia Geral, CCB, Universidade Estadual de Londrina, P.O Box 6001, Londrina, Paraná CEP 86051-970, Brazil

**Keywords:** Fluorochromes, heterochromatin, karyotype evolution, pisces, rDNA

## Abstract

The family Curimatidae is a fish group usually considered chromosomally conserved in their diploid number. However, some studies show small changes in the karyotype microstructure, and the presence of B chromosomes, indicating a chromosomal diversification within the group, even if structural changes in the karyotypes are not visible. Few studies associate this trait with an evolutionary pattern within the family. This study aimed to characterize the karyotype, nucleolus organizer regions (NORs), and heterochromatin distribution of six species of Curimatidae of the genera *Cyphocharax* Fowler, 1906 and *Steindachnerina* Fowler, 1906: *Cyphocharax voga* (Hensel, 1870), *Cyphocharax spilotus* (Vari, 1987), *Cyphocharax saladensis* (Meinken, 1933), *Cyphocharax modestus* (Fernández-Yépez, 1948), *Steindachnerina biornata* (Braga et Azpelicueta, 1987) and *Steindachnerina insculpta* (Fernández-Yépez, 1948) and contribute data to a better understanding of the mechanisms involved in the chromosomal evolution of this group of fish. All specimens had 2n=54, m-sm, and B microchromosomes. Five species exhibited single NORs, except for *Steindachnerina biornata*, which showed a multiple pattern of ribosomal sites. NORs were chromomycin A_3_ positive (CMA_3_^+^) and 4’-6-diamino-2-phenylindole (DAPI^-^) negative, exhibiting differences in the pair and chromosomal location of each individual of the species. FISH with 5S rDNA probe revealed sites in the pericentrometic position of a pair of chromosomes of five species. However, another site was detected on a metacentric chromosome of *Cyphocharax spilotus*. Heterochromatin distributed both in the pericentromeric and some terminal regions was revealed to be CMA_3_^+^/DAPI^-^. These data associated with the previously existing ones confirm that, although Curimatidae have a very conservative karyotype macrostructure, NORs and heterochromatin variability are caused by mechanisms of chromosome alterations, such as translocations and/or inversions, leading to the evolution and diversification of this group of fish.

nucleolus organizer regions

## Introduction

Cytogenetic studies in Neotropical fish reveal great chromosome diversity with both intra- and interspecific karyotype variability. Within the order Characiformes, there are two distinct trends: groups that show a significant difference in diploid number and/or karyotype formulae and karyotypically homogeneous groups (Galetti et al. 1994). Given these trends, the family Curimatidae belongs to the second group. Of the 101 described species (Netto-Ferreira et al. 2011), 38 have been cytogenetically assessed. The studies revealed that 32 of latter exhibited a diploid number (2n) of 54 chromosomes and a fundamental number (FN) equal to 108 (Sampaio et al. 2011).

Small changes in the karyotype microstructure involving the nucleolus organizer regions (NORs) and heterochromatin distribution pattern occur as a result of chromosomal evolution. Such alterations can be regarded as relevant cytogenetic markers. Consequently, despite being considered conserved, some species of this group present exceptions to the observed regularity, allowing inferences about the evolutionary pathways within the family (Galetti Jr. et al. 1994; Galetti Jr. 1998).

Another feature considered a chromosomal diversification within Curimatidae is the presence of B chromosomes in some species (Vênere et al. 2008). This chromosome, also called supernumerary or accessory, may exhibit either a similar morphology to that of the chromosomes of the A complement, or one that is to a clearly distinct. The number of Bs may vary among the different cells of the same individual in species that possess them. This variation may be ascribable to an anaphasic delay, with the removal of B from some cells or tissues, or to meiotic nondisjunction, when both chromatids migrate to the same pole (Camacho et al. 2000). Hitherto, B chromosomes have been described in seven species of Curimatidae of different populations: *Cyphocharax gouldingi* Vari, 1992, *Cyphocharax modestus* (Fernández-Yépez, 1948), *Cyphocharax saladensis* (Meinken, 1933), *Cyphocharax spilotus* (Vari, 1987), *Cyphocharax voga* (Hensel, 1870), *Steindachnerina biornata* (Braga & Azpelicueta, 1987) and *Steindachnerina insculpta* (Fernández-Yépez, 1948) (Sampaio et al. 2011; Vênere et al. 2008).

Although a number of cytogenetic studies show conservation of the diploid number (2n=54) in the family Curimatidae, divergence of nucleolus organizer regions and C-banding was observed. Nevertheless, few studies correlate the cytogenetic characteristics to the evolutionary trends within the family. Thus, this study aimed to characterize the karyotype, nucleolus organizer regions (NORs), and heterochromatin distribution of six species of Curimatidae of the genera *Cyphocharax* Fowler, 1906 and *Steindachnerina* Fowler, 1906, as well as contribute to a better understanding of the mechanisms underlying the chromosomal evolution of this interesting group of fish.

## Materials and methods

### Collection sites

Six species of the family Curimatidae were analysed: *Cyphocharax voga*, *Cyphocharax spilotus*, *Cyphocharax saladensis*, *Cyphocharax modestus*, *Steindachnerina biornata* and *Steindachnerina insculpta*, collected from the Laguna dos Patos Hydrographic System/RS, Tramandaí River basin/RS, and Paranapanema River basin/SP/PR (Table 1). Voucher specimens are catalogued in the Zoology Museum of the Universidade Estadual de Londrina, Paraná, under catalog numbers: MZUEL 1374 - *Cyphocharax modestus*; MZUEL 5058 - *Cyphocharax saladensis*; MZUEL 5106 - *Cyphocharax spilotus*; MZUEL 5105 - *Cyphocharax voga*; MZUEL 5059 - *Steindachnerina biornata*; MZUEL 1042 - *Steindachnerina insculpta*.

**Table 1.** Species, collection sites and hydrographic basins.

Species	Number of individuals	Collection sites	Hydrographic basin
*Cyphocharax modestus*	5♀, 6♂	Três Bocas stream, Londrina, PR, Brazil S 23°17'12.9" W 51°13'58.2"	Paranapanema river
*Cyphocharax saladensis*	1♀, 9♂	Agronomic Experiment Station of UFRGS’s Dam, Eldorado do Sul, RS, Brazil S 30°05'33.7" W 51°40'40.0"	Laguna dos Patos hydrographic system
*Cyphocharax spilotus*	2♀, 2♂	Capivara stream, Barra do Ribeiro, RS, Brazil S 30°17'34.0" W 51°19'21.2"	
	1♂	Gasômetro, Porto Alegre, RS, Brazil S 30°02'06.3" W 51°14'29.12"	
*Cyphocharax voga*	1♀, 1♂	Saco da Alemoa river, Eldorado do Sul, RS, Brazil S 29°59'15.6" W 51°14'24.1"	
	3♀, 9♂	Capivara stream, Barra do Ribeiro, RS, Brazil S 30°17'34.0" W 51°19'21.2"	
	1♀, 3♂	Gasômetro, Porto Alegre, RS, Brazil S 30°02'06.3" W 51°14'29.12"	
	5♂	Barros lagoon, Osório, RS, Brazil S 29°56'30.0" W 50°19'32.0"	
	3♀, 4♂	Quadros lagoon – Barra do João Pedro, Maquiné, RS, Brazil S 29°46'21.2" W 50°05'08.0"	Tramandaí river
*Steindachnerina biornata*	1♀, 1♂	Forquetinha river, Canudos do Vale, RS, Brazil S 29°24'22.4" W 52°03'19.2"	Laguna dos Patos hydrographic system
*Steindachnerina insculpta*	3♀, 2♂	Três Bocas stream, Londrina, PR, Brazil S 23°17'12.9" W 51°13'58.2"	Paranapanema river
	2♂	Pavão stream, Sertanópolis, PR, Brazil	
	6♀, 12♂	Jacutinga river, Londrina, PR, Brazil S 23°23'6.6" W 51°04'35.8"	
	3♀, 7♂	Água dos Patos river, Iepê, SP, Brazil S 23°12'23.3" W 50°56'49.1"	
Total of individuals:	93		

### Conventional staining

Mitosis was stimulated by injecting animals with a yeast suspension (Lee and Elder 1980). Mitotic chromosomes were obtained by direct preparation, removing the anterior kidney, with hypotonic treatment, methanol:acetic acid fixation and air-drying (Bertollo et al. 1978). Lastly, the chromosomes were stained with 5% Giemsa in phosphate buffer (pH 6.8), and classified as metacentric (m) and submetacentric (sm) (Levan et al. 1964).

### Chromosome Banding

The distribution of heterochromatin was analyzed by C-banding (Sumner 1972). Silver nitrate staining of the active nucleolus organizer regions (AgNOR) was performed according to Howel and Black (1980). The GC and AT-rich bands were detected using Chromomycin A3 (CMA3) and 4’,6-diamidino-2-phenylindole (DAPI), respectively, according to Schweizer (1980).

### Fluorescence in situ hybridization


 Fluorescence *in situ* hybridization (FISH) followed the methods described by Pinkel et al. (1986) with an 18S rDNA probe obtained from *Prochilodus argenteus* Spix & Agassiz, 1829 (Hatanaka and Galetti Jr. 2004). The 18S rDNA probe was labeled with biotin-14-dATP (Roche Applied Science) by nick translation and the 5S rDNA probe from *Leporinus elongatus* Linnaeus, 1758 (Martins and Galetti Jr. 2001) was labeled with digoxigenin 11-dUTP (Roche Applied Science) by PCR. The hybridization signal was detected using avidin-FITC (fluorescein isothiocyanate) (Life Technologies) for the 18S rDNA probe and anti-digoxigenin-rhodamine (Roche Applied Science) for the 5S rDNA probe. The chromosomes were counterstained with propidium iodide or DAPI, respectively. All the images were acquired with a Leica DM 4500 B microscope equipped with a DFC 300FX camera and Leica IM50 4.0 software and optimized for best constrast and brightness with Adobe Photoshop CS6 software.

## Results

**Figure 1.**
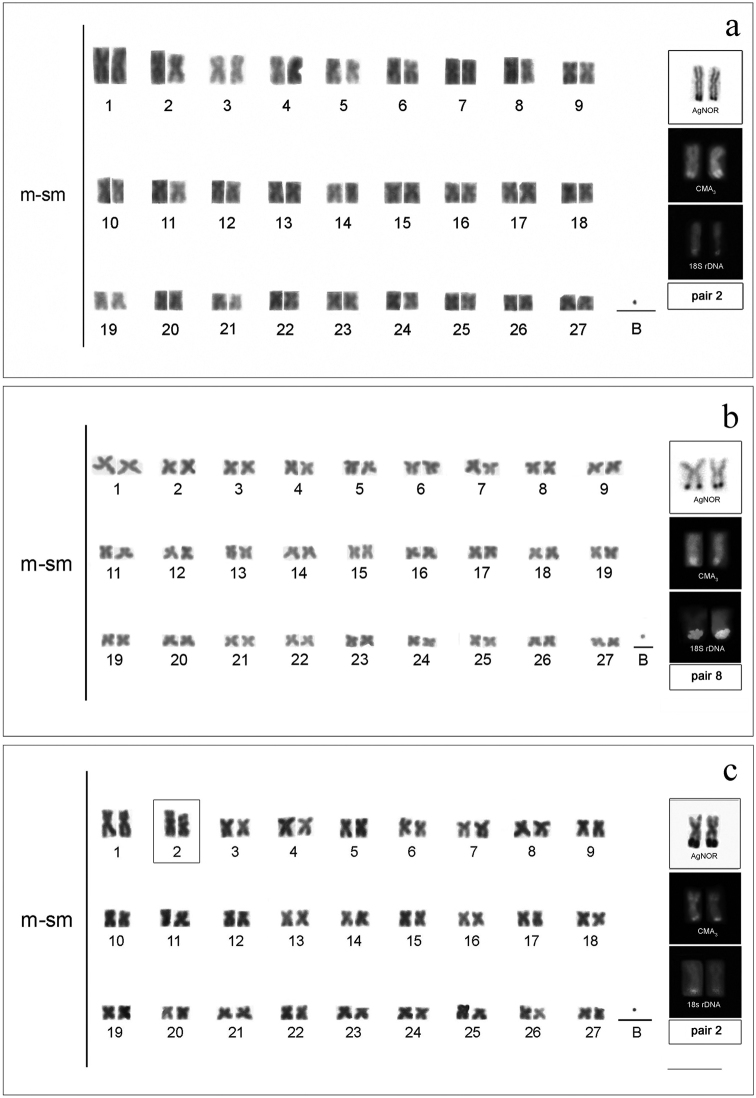
Karyotypes with B microchromosome of: **a** *Cyphocharax modestus* **b** *Cyphocharax saladensis* **c** *Cyphocharax spilotus*, showing AgNORs, CMA_3_ and 18S rDNA sites of each species. Note the secondary constrictions in square box (**c**). Bar: 5 µm.

**Figure 2.**
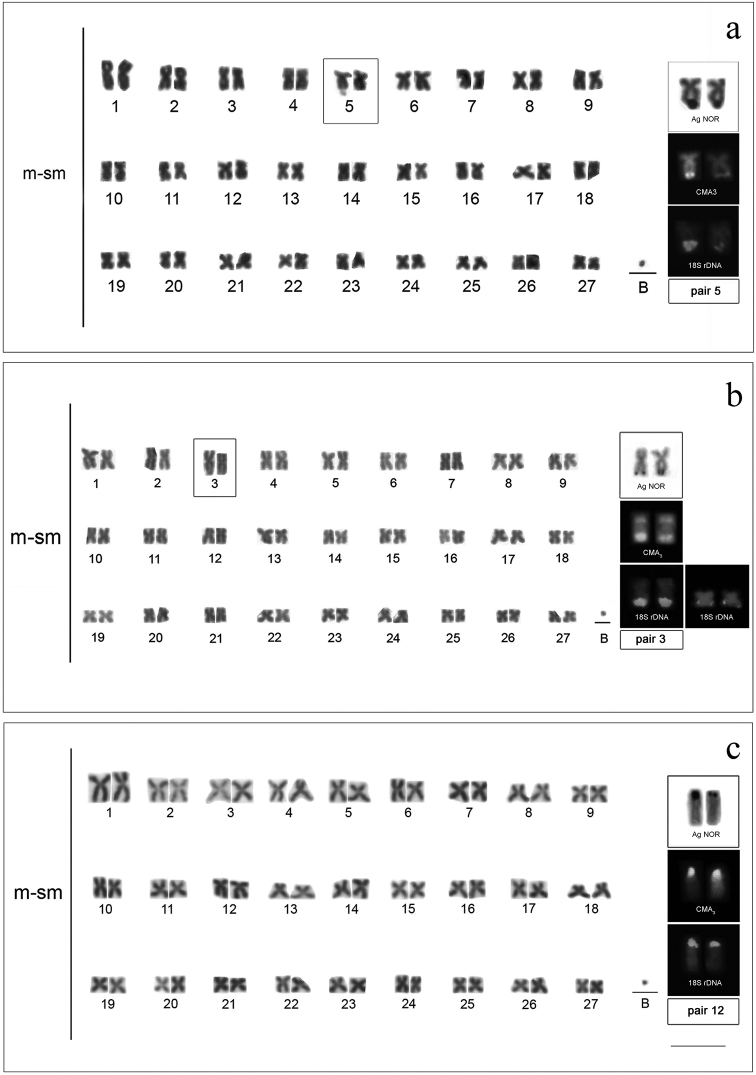
Karyotypes with B microchromosome of: **a** *Cyphocharax voga* **b** *Steindachnerina biornata* **c** *Steindachnerina insculpta*, showing AgNORs, CMA_3_ and 18S rDNA sites of each species. Note the secondary constrictions in square box (**a**, **b**). Bar: 5 µm

All species analyzed showed 54 meta-submetacentric chromosomes (m-sm) and fundamental number (FN) equal to 108. All populations presented individuals with B microchromosomes of a dot type in all somatic cells (Figs 1, 2). Terminal secondary constrictions occurred in *Cyphocharax voga* and *Steindachnerina biornata*, on the long arm of pairs 5 and 3, respectively (Figs 2a, b, box), and in the interstitial position of *Cyphocharax spilotus*, on the short arm of the second pair (Fig. 1c, box).

One AgNOR was observed in the terminal region of a pair of chromosomes in all species (Figs 1, 2, box). Table 2 shows the pair and the position of this region in each species. The secondary constriction was coincident with the AgNOR in *Cyphocharax voga* (pair 5) and *Steindachnerina biornata* (pair 3) (Figs 2a, b, box). In *Cyphocharax spilotus*, the AgNOR was located in the terminal position on the long arm of pair 2, and was not coincident with the interstitial constriction on the short arm of this same pair (Fig. 1c, box).

**Table 2.** Chromosome pairs and positions of the nucleolus organizer regions (AgNORs).

Species	AgNOR pair	AgNOR position on chromosome	Secondary constriction
*Cyphocharax modestus*	02	Terminal/long arm	------
*Cyphocharax saladensis*	08	Terminal/long arm	------
*Cyphocharax spilotus*	02	Terminal/long arm	Interstitial/short arm
*Cyphocharax voga*	05	Terminal/long arm	Terminal/long arm
*Steindachnerina biornata*	03	Terminal/long arm	Terminal/long arm
*Steindachnerina insculpta*	12	Terminal/short arm	------

The AgNORs in the species *Cyphocharax modestus*, *Cyphocharax saladensis*, *Cyphocharax spilotus*, *Cyphocharax voga*, and *Steindachnerina insculpta* were confirmed by fluorescence *in situ* hybridization (FISH) using an 18S rDNA probe (Figs 1, 2, box). *Steindachnerina biornata* presented a small pair of metacentric chromosomes with 18S ribosomal sites in the terminal region of the long arm, besides the pair impregnated with silver (Fig. 2b, box). Staining with CMA_3_ fluorochromes revealed fluorescent signals in the terminal region of a chromosome pair corresponding to the AgNORs in all species (Figs 1, 2, box).

**Figure 3.**
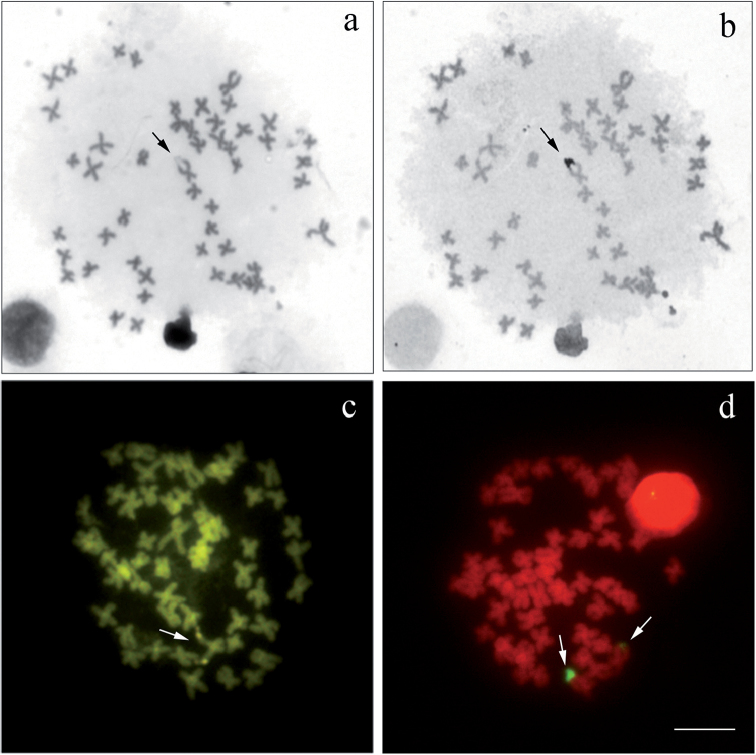
Metaphases of *Cyphocharax voga* (Barros lagoon/RS): **a** Giemsa **b** AgNOR (sequential) **c** CMA_3_ **d** 18S rDNA FISH. The arrows indicate the chromosome carrying the secondary constriction and AgNOR. Bar: 5 µm.

Two individuals of *Cyphocharax voga* collected in the Lagoa dos Barros/RS showed a block corresponding to the AgNOR and the CMA_3_ fluorochrome on the secondary constriction of a chromosome. FISH revealed two chromosomes with terminal 18S rDNA sites. One of the sites was larger than the other, revealing heteromorphism of this region (Fig. 3).

**Figure 4.**
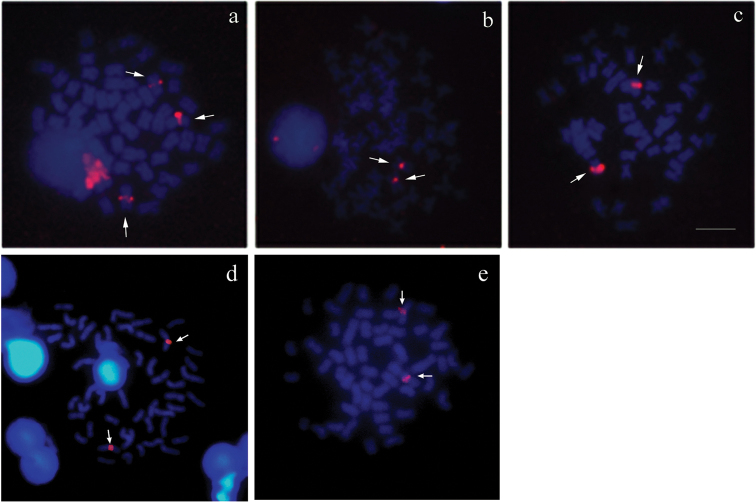
5S rDNA FISH of: **a** *Cyphocharax spilotus* **b** *Cyphocharax voga* **c** *Steindachnerina insculpta* **d** *Cyphocharax modestus* **e** *Cyphocharax saladensis*. Note in (**a**) the presence of a small chromosome of *Cyphocharax spilotus* with 5S rDNA sites (arrowhead). Bar: 5 µm.

FISH with a 5S rDNA probe revealed sites in the pericentromeric position of a pair of metacentric chromosomes of five species: *Cyphocharax spilotus*, *Cyphocharax voga*, *Steindachnerina insculpta*, *Cyphocharax modestus* and *Cyphocharax saladensis*. Furthermore, another site was detected on a smaller metacentric chromosome of *Cyphocharax spilotus* (Fig. 4). These regions did not coincide with the 18S rDNA site. In *Steindachnerina biornata*, we could not obtain favorable results with the 5S rDNA probe.

**Figure 5.**
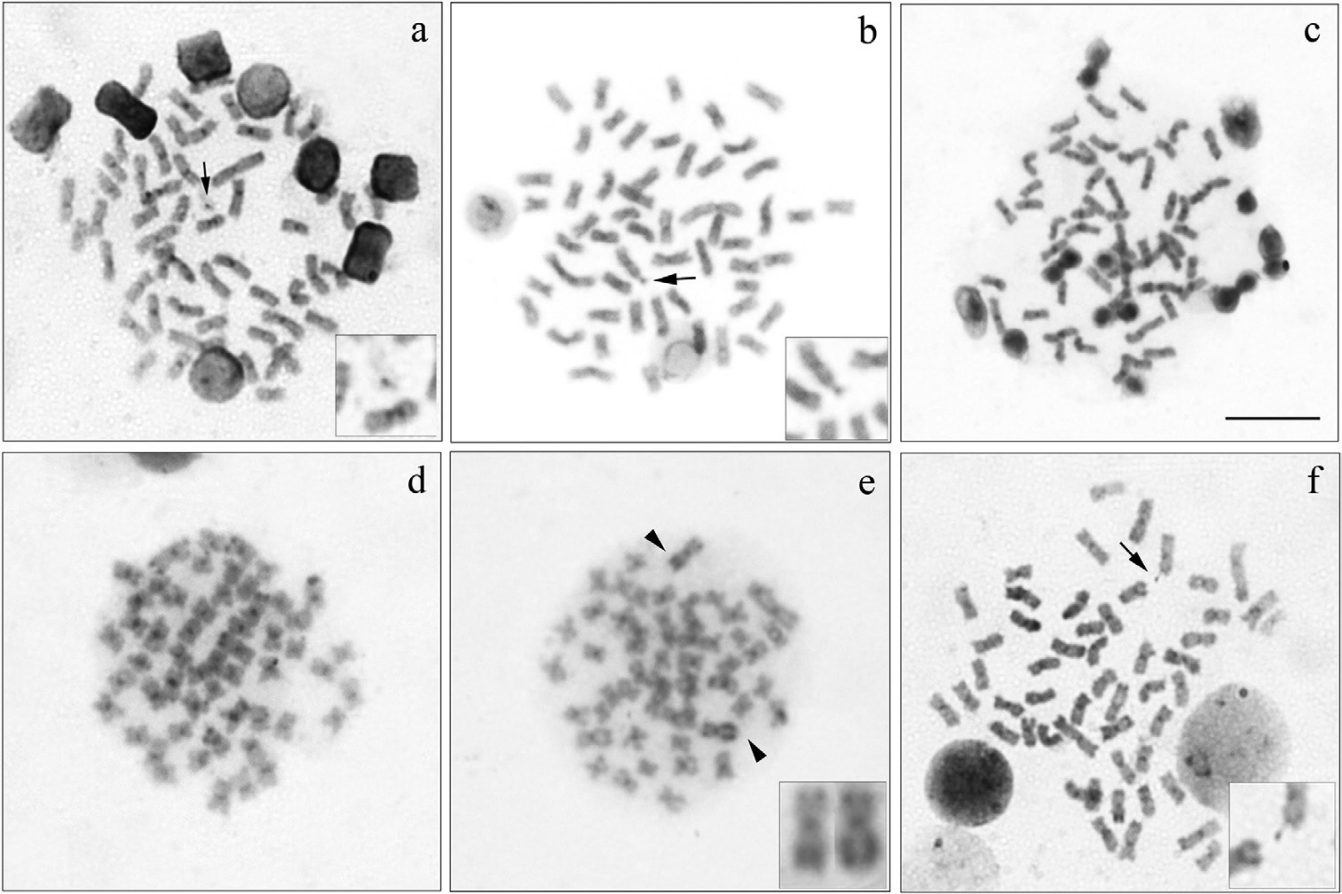
Metaphases with C-banding of: **a** *Cyphocharax modestus* **b** *Cyphocharax saladensis* **c** *Cyphocharax spilotus* **d** *Cyphocharax voga* **e** *Steindachnerina biornata* **f** *Steindachnerina insculpta*. Arrows and square box in (**a**), (**b**) and (**f**) highlight the heterochromatic B microchromosome. Note in (**e**) the pair of *Steindachnerina biornata* with terminal heterochromatic regions on the long and short arm. Bar: 5 µm.

**Figure 6.**
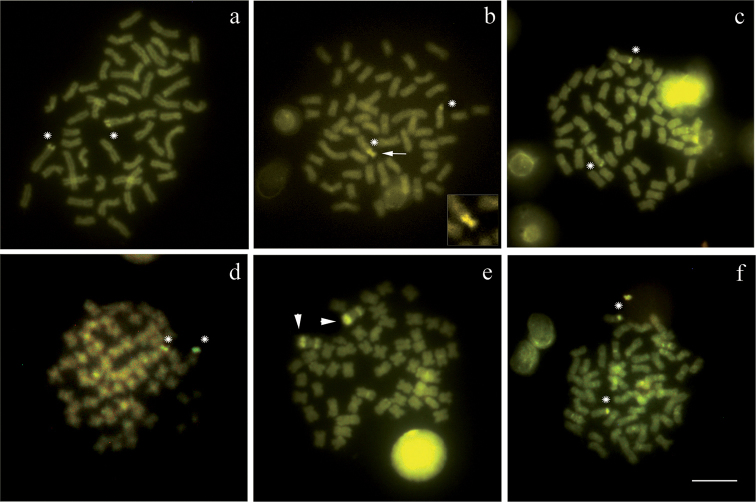
Metaphases with C-banding staining with CMA_3_ of: (**a**) *Cyphocharax modestus*; (**b**) *Cyphocharax saladensis*; (**c**) *Cyphocharax spilotus*; (**d**) *Cyphocharax voga*; (**e**) *Steindachnerina biornata*; (f) *Steindachnerina insculpta*. The (*) indicates the NOR pairs. Note in (**b**) the heterochromatic CMA_3_^+^ B microchromosome of *Cyphocharax saladensis* (arrow and square box) and in (**e**) the heterochromatic pair of *Steindachnerina biornata* (arrowhead). Bar: 5 µm.

Heterochromatin in Curimatidae species was preferentially observed in the pericentromeric and some terminal regions (Fig. 5). After fluorochrome staining, all heterochromatic regions proved CMA_3_^+^ (Figure 6). *Steindachnerina biornata* exhibited heterochromatin in the two terminal regions of the NOR-bearing pair, namely one block on the long arm and a discrete marking on the short arm. After CMA_3_ fluorochrome staining, these areas became fluorescent (Figs 5e, 6e).

Microchromosome B proved to be heterochromatic in *Cyphocharax modestus*, *Cyphocharax saladensis*, and *Steindachnerina insculpta* (Figures 5a, b, f box, respectively). Its visualization with C-banding was not possible in the other species. Only in *Cyphocharax saladensis*, the heterochromatic fluorescent B chromosome was observed after staining with CMA_3_ fluorochrome (Figure 6b).

## Discussion

This study showed the first chromosome banding data for populations of Curimatidae of the Lagoa dos Patos and Tramandaí River basins, in the state of Rio Grande do Sul, as well as the first data on the species *Cyphocharax saladensis* and *Steindachnerina biornata*. All species maintained the pattern, presenting 2n = 54 m-sm. The model proposed by Feldberg et al. (1992), corroborates that this is an ancestral karyotype of Curimatidae and that variations of this condition represent derived characters. Considering Feldberg’s assertions, it is possible to affirm that concerning the karyotype macrostructure, the Curimatidae species studied herein have basal karyotypes. The presence of basal karyotypes is common in this group. However, Brassesco et al. (2004), found variations in the diploid number of *Cyphocharax platanus* (Günther, 1880), which showed a 2n = 58 and karyotype formula of 52m-sm+6st and *Potamorhina squamoralevis* (Braga & Azpelicueta, 1983), which had 2n = 102 and 14m-sm +88a. These data indicate that the chromosomal evolution in some species of Curimatidae is followed by alterations as centric fissions and inversions in the karyotype macrostructure (Feldberg et al. 1993; Brassesco et al. 2004).

Sampaio et al. (2011) analyzed the mitotic and meiotic behavior of B microchromosomes in the species assessed herein, corroborating that this is an important cytogenetic characteristic in this group of fish. Currently, the occurrence of these B chromosomes has been reported in seven species of Curimatidae from different populations, corresponding to 18.42% of the total studied species (Sampaio et al. 2011). Although considered a remarkable feature in the Curimatidae family, only 2 of the 8 genera analyzed, i.e., *Cyphocharax* and *Steindachnerina*, have presented this type of chromosome thus far (Table 3).

**Table 3.** Chromosome studies in the family Curimatidae. *2n*, diploid number; *FN*, fundamental number; *m*, metacentric; *sm*, submetacentric; *st*, subtelocentric; *a*, acrocentric; *B*, supernumerary chromosome; *term*., terminal; *peric*., pericentromeric; *centr*., centromeric; *inters*., interstitial.

Species	Locality	2n	Karyotypic formula	FN	AgNOR pair	Position	Number of cistrons 18S rDNA	Number/position of cistrons 5S rDNA	C banding	Reference
*Curimata cyprinoides*	Negro and Solimões river/AM	54	44m + 10sm	108	3	term. long arm	-	-	-	3
	Araguaia river/MT	54	44m + 10sm	108	7	term. long arm	-	-	-	16
*Curimata inornata*	Negro and Solimões river/AM	54	40m + 14sm	108	21	inters. short arm	-	-	-	3
	Araguaia river/MT	54	40m + 14sm	108	3, 22	term. long arm	-	-	Peric./term.	16
*Curimata kneri*	Negro and Solimões river/AM	54	40m + 14sm	108	27	term. short arm	-	-	-	3
*Curimata ocellata*	Negro and Solimões river/AM	56	40m + 16sm	112	26	inters. short arm	-	-	-	3
*Curimata vittata*	Negro and Solimões river/AM	54	42m + 12sm	108	9	term. long arm	-	-	-	3
*Curimatella alburna*	Negro and Solimões river/AM	54	46m + 8sm	108	14	term. long arm	-	-	-	3
*Curimatella dorsalis*	Miranda river/MS	54	46m + 8sm	108	13	term. short arm	-	-	Peric.	7
	Paraná river/AR	54	54m/sm	108	2	term. long arm	-	-	Centr./term.	11
*Curimatella imaculata*	Araguaia river/GO	54	46m + 8sm	108	24	inters. long arm	-	-	Peric.	16
*Curimatella lepidura*	São Francisco river/SP	54	54m/sm	108	9	term. short arm	-	-	-	2
*Curimatella meyeri*	Negro and Solimões river/AM	54	46m + 8sm	108	9	term. long arm	-	-	-	3
*Curimatopsis myersi*	Miranda river/MS	46	42m + 4sm	92	-	-	-	-	-	7
*Cyphocharax gilbert*	Paraibuna river/SP	54	44m + 10sm	108	2	term. short arm	-	-	Peric./term.	16
Cyphocharax cf. gillii	Bento Gomes river/MT	54	54m/sm	108	1	inters. long arm	-	-	-	2
*Cyphocharax gouldingi*	Araguaia river/GO	54	54m + B	108	2	term. long arm	-	-	Peric.	16
*Cyphocharax modestus*	Tiête river/SP	54	54m/sm/B	108	-	term. long arm	-	-	Centr./term.	1
	Águas de São Pedro/SP	54	54m/sm	108	2	term. long arm	-	-	-	2
	Três Bocas stream/PR	54	54m/sm + B	108	2	term. long arm	2	-	Peric./term.	6, 13, 15, 18, 19
	Mogi-Guaçu river/SP	54	54m/sm + B	108	-	-	-	-	Peric.	8
	Taquari river/PR	54	54m/sm + B	108	2	term. long arm	2	-	Peric./term.	13, 15
	Tibagi river/PR	54	54m/sm	108	2	term. long arm	2	-	-	15
	Água da Floresta river/PR	54	54m/sm	108	2	term. long arm	2	-	-	15
	Paranapanema river/SP	54	54m/sm + B	108	2	term. long arm	2	4/peric. short arm	Centr./term.	12, 14, 17
	Tietê river/SP	54	54m/sm	108	2	term. long arm	2	4/peric. short arm	Centr./term.	12, 14, 17
									
*Cyphocharax nagelii*	Mogi-Guaçu river/SP	54	54m/sm	108	25	term. short arm	-	-	-	2
	Mogi-Guaçu river/SP	54	46m + 8sm	108	1, 2, 6, 11, 21	term. long /short arm	-	-	Peric./term.	16
*Cyphocharax platanus*	Paraná river/AR	58	52m/sm + 6st	116	5	term. short arm	-	-	Centr.	11
	Pirá-Pytá stream/ AR	58	48m + 4 sm + 6st	116	6	term. short arm	-	-	Peric./term.	16
Cyphocharax cf. spilurus	Madeira river/RO	54	54m/sm	108	10	term. long arm	-	-	-	2
*Cyphocharax spilotus*	Paraná river/AR	54	54m/sm + B	108	1	inters. long arm	-	-	Centr./term.	10, 11
	Capivara stream/RS	54	54m/sm + B	108	2	term. long arm	2	-	Peric./term.	18, 19
	Gasômetro/RS	54	54m/sm + B	108	2	term. long arm	2	3/peric. short arm	Peric./term.	18, 19
*Cyphocharax vanderi*	Preto river/SP	54	54m/sm	108	6	term. long arm	-	-	-	2
*Cyphocharax voga*	Bolacha stream/RS	54	54m/sm	108	6	term. long arm	-	-	-	2
	Paraná river/AR	54	54m/sm	108	-	term. long arm	-	-	Inters./term.	11
	Saco da Alemoa river/RS	54	54m/sm + B	108	5	term. long arm	2	-	Peric./term.	18, 19
	Capivara stream/RS	54	54m/sm + B	108	5	term. long arm	2	-	Peric./term.	18, 19
	Gasômetro/RS	54	54m/sm + B	108	5	term. long arm	2	-	Peric./term.	18, 19
	Barros lagoon/RS	54	54m/sm + B	108	5	term. long arm	2	2/peric. short arm	Peric./term.	18, 19
	Quadros lagoon/RS	54	54m/sm + B	108	5	term. long arm	2	-	Peric./term.	18, 19
*Cyphocharax saladensis*	A.E.S UFRGS dam/RS	54	54m/sm + B	108	8	term. long arm	2	2/peric. short arm	Peric./term.	18, 19
*Potamorhina altamazonica*	Negro and Solimões river/AM	102	2m + 2sm + 98a	106	5	term. long arm	-	-	Peric./inters/term.	4
*Potamorhina latior*	Negro and Solimões river/AM	56	52m + 2sm + 2st	112	25	term. long arm	-	-	Peric./term.	4
*Potamorhina pristigaster*	Negro and Solimões river/AM	54	42m + 12sm	108	25	term. short arm	-	-	Peric.	4
*Potamorhina squamoralevis*	Paraná river/AR	102	14m/sm + 88a	116	-	term. long arm	-	-	Centr.	11
*Psectrogaster amazonica*	Araguaia river/MT	54	44m + 10sm	108	17	term. short arm	-	-	Peric.	16
*Psectrogaster curviventris*	Miranda river/MS	54	42m + 12sm	108	20	term. short arm	-	-	Peric.	7
	Paraná river/AR	54	54m/sm	108	-	inters. long arm	-	-	Centr./term.	11
*Psectrogaster rutiloides*	Negro and Solimões river/AM	54	42m + 12sm	108	9	term. long arm	-	-	-	3
*Steindachnerina amazonica*	Araguaia river/GO	54	42m + 12sm	108	2, 23	term. long arm	-	-	Peric./term.	16
*Steindachnerina biornata*	Forquetinha river/RS	54	54m/sm + B	108	3	term. long arm	4	-	Peric./term.	18, 19
									
*Steindachnerina brevipinna*	Miranda river/MS	54	48m + 6sm	108	17	term. short arm	-	-	Centr./term.	7
	Paraná river/AR	54	54m/sm	108	15	term. long arm	-	-	Centr./inters./term.	11
*Steindachnerina conspersa*	Paraguai river/MS	54	54m/sm	108	2	inters. long arm	-	-	-	2
	Paraná river/AR	54	54m/sm	108	2	term. long arm	-	-	Centr./inters/term.	11
*Steindachnerina elegans*	São Francisco river/SP	54	54m/sm	108	25	term. short arm	-	-	-	2
*Steindachnerina gracilis*	Araguaia river/MT	54	38m + 16sm	108	-	term. long arm	-	-	Peric.	16
Steindachnerina cf. guentheri	São Francisco river/AC	54	54m/sm	108	24	term. short arm	-	-	Peric./inters/term.	9
*Steindachnerina insculpta*	Mogi-Guaçu river/SP	54	54m/sm	108	25	term. short arm	-	-	-	2
	Passa-Cinco river/SP	54	54m/sm	108	25	term. short arm	-	-	-	2
	Paranapanema river/SP	54	54m/sm + B	108	-	-	-	-	Peric.	5
	Reserva Jurumirim/SP	54	54m/sm + B	108	-	-	-	-	Peric.	5
	Paranapanema river/SP	54	54m/sm	108	7	term. short arm	2	2/peric. short arm	Centr./term.	12, 14, 17
	Tietê river/SP	54	54m/sm	108	7	term. short arm	2	2/peric. short arm	Centr./term.	12, 14, 17
	Três Bocas stream/PR	54	54m/sm + B	108	7	term. short arm	2	-	Peric./term.	13, 15
	Taquari river/PR	54	54m/sm	108	7	term. short arm	2	-	Peric./term.	13, 15
	Tibagi river/PR	54	54m/sm	108	7	term. short arm	2	-	Peric./term.	13, 15
	Água da Floresta river/PR	54	54m/sm	108	7	term. short arm	2	-	Peric./term.	13, 15
	Cachoeira de Emas/SP	54	54m/sm	108	22	term. short arm	-	-	Peric./term.	16
	Água dos Patos river/SP	54	54m/sm + B	108	12	term. short arm	2	-	Peric./term.	18, 19
	Três Bocas streams/PR	54	54m/sm + B	108	12	term. short arm	2	2/peric. short arm	Peric./term.	18, 19
	Pavão stream/PR	54	54m/sm + B	108	12	term. short arm	2	-	Peric./term.	18, 19
	Jacutinga river/PR	54	54m/sm + B	108	12	term. short arm	2	-	Peric./term.	18, 19
*Steindachnerina leucisca*	Negro and Solimões river/AM	54	48m + 6sm	108	15	term. short arm	-	-	-	3

**1.** Venere and Galetti (1985); **2.** Venere and Galetti (1989); **3.** Feldberg et al. (1992); **4.** Feldberg et al. (1993); **5.** Oliveira and Foresti (1993); **6.** Martins et al. (1996); **7.** Navarrete and Júlio-Jr. (1997); **8.** Venere et al. (1999); **9.** Carvalho et al. (2001); **10.** Fenocchio et al. (2003); **11.** Brassesco et al. (2004); **12.** De Rosa et al. (2006); **13.** Gravena et al. (2007); **14.** De Rosa et al. (2007); **15.** Teribele et al. (2008); **16.** Venere et al. (2008); **17.** De Rosa et al. (2008); **18.** Sampaio et al. (2011); **19.** present paper.

Besides the presence of B chromosomes, another striking feature of the Curimatidae species are the nucleolus organizer regions. Previous works have described the AgNORs of *Cyphocharax spilotus* and *Steindachnerina insculpta* on other pairs besides those observed here (Table 3), showing an interpopulation variability in the location of AgNORs among Curimatidae. These fish occur in different ecosystems of the Neotropical region, and isolated populations can be established under different environmental conditions, enabling an increase in the frequency of certain variations (Brassesco et al. 2004; Vari 2003). These variations may be ascribable to rearrangements of the chromosomal microstructure, such as translocations and/or inversions (Venere and Galetti Jr. 1989; De Rosa et al. 2007).

All studied populations of *Cyphocharax modestus* presented the AgNOR on pair 2. The populations of *Cyphocharax voga* presented the AgNOR mainly on pair 5 (Table 3), indicating that these sites can be considered important species-specific cytogenetic markers (Venere et al. 2008; De Rosa et al. 2007).

In many fish groups, including Curimatidae, there is a high correlation between AgNORs and secondary constriction (Feldberg et al. 1992; Teribele et al. 2008; Gouveia et al. 2013). However, the presence of secondary constriction without rDNA sequences, as in *Cyphocharax spilotus*, is a characteristic rarely observed in fish. But this can occur due to the existence of pseudo-NORs, appearing decondensed and stained with silver nitrate, being transcriptionally inactive (Prieto and McStay 2008).

The results of FISH in *Steindachnerina biornata* showed another species with multiple NOR patterns among Curimatidae. The above method revealed an unusual feature, which was observed only in *Curimata inornata* Vari, 1989, *Cyphocharax nagelii* (Steindachner, 1881), *Steindachnerina amazonica* (Steindachner, 1911), and *Steindachnerina gracilis* Vari & Vari, 1989 (Venere et al. 2008). As shown in Table 3, most studies with NORs have utilized only silver nitrate, which may explain the small number of species with multiple sites in this group of fish.

The existing literature presents scarce data on fluorochrome staining in the family Curimatidae, with reports only in *Cyphocharax modestus* and *Steindachnerina insculpta* (De Rosa et al. 2007; Teribele et al. 2008; Martins et al. 1996) and the results are coincident with those observed in this study, indicating that NORs are rich in GC base pairs.

NOR heteromorphism in the homologous chromosomes of two individuals of *Cyphocharax voga* from the population of the Lagoa dos Barros/RS may be attributable to unequal crossing over, where the small site may have become inactive, or could not be detected by silver nitrate or CMA_3_ because of their size. Teribele et al. (2008), obtained similar results in an individual of *Cyphocharax modestus* collected in the Taquari River/PR.

FISH with the 5S rDNA probe revealed results coincident with those found by Da Rosa et al. (2006) in studies on the *Cyphocharax modestus* and *Steindachnerina insculpta*, which also showed ribosomal sites in the pericentromeric region of a chromosome pair, suggesting the existence of homology between these species. These authors observed smaller signals on a second pair of chromosomes in *Cyphocharax modestus*, similar to the small 5S rDNA site found on the single metacentric chromosome in *Cyphocharax spilotus*.

To explain the presence of larger and smaller 5S rDNA sites, De Rosa et al. (2006), compared Curimatidae with other families comprising species with the same behavior sequences, such as *Leporinus* Agassiz, 1829 and *Schizodon* Agassiz, 1829 (Anostomidae), *Parodon* Valenciennes, 1850 (Parodontidae) and *Prochilodus argenteus* Spix & Agassiz, 1829 (Prochilodontidae). These families, along with Curimatidae, form a monophyletic group based on morphological characteristics showing that their 5S rDNA clusters have possibly been preserved from significant changes during the evolution.

C-banding analyses did not allow us to characterize and differentiate among the species and/or genera analyzed in this study. However, Venere et al. (2008) observed a pronounced diversification in the distribution and amount of heterochromatin in some species of Curimatidae, differentiating between the genera *Steindachnerina* and *Cyphocharax*, indicating the heterochromatin characterization in chromosomes of each group.

The difference in the amount of heterochromatin in Curimatidae reflects the interpopulation variability occurring within this family. It is believed that the amount of heterochromatin can play a significant role in the chromosome evolution in this fish group. As previously mentioned, Curimatidae can be established in isolated populations under different environmental conditions. Such conditions may enable increased variations in the distribution of heterochromatin.

CMA_3_ fluorochrome staining revealed fluorescent signals in the heterochromatic regions of many chromosomes of the complement, showing that heterochromatin in these species consists mostly of GC base pairs. A chromosomal pair detected in *Steindachnerina biornata* can be considered a species-specific marker, since we evidenced heterochromatin in the two terminal regions of the NOR-bearing pair, i.e., a block on the long arm associated with the NOR and a more discreet marking on the short arm. The NOR adjacent to the heterochromatic blocks may facilitate chromosome breakage, leading to structural rearrangements in these regions (Moreira-Filho et al. 1984).

In *Cyphocharax modestus*, *Cyphocharax saladensis*, and *Steindachnerina insculpta*, the B microchromosome presented itself entirely heterochromatic, indicating the total absence of gene activity, as in other studied populations of *Cyphocharax modestus* (Gravena et al. 2007; Venere et al. 1999) and *Steindachnerina insculpta* (Gravena et al. 2007). The heterochromatic B chromosome of *Cyphocharax saladensis* proved CMA_3_^+^, therefore, rich in GC base pairs.

Two hypotheses have been proposed for the origin of B chromosomes in Curimatidae (Martins et al. 1996). The first suggests a common B chromosome ancestor, which may have arisen in the ancestors of the family, and eliminated from the present species that do not have B-chromosome. The second proposes that B chromosomes may have had a recent and independent origin, resulting in closely related species, or even in the same species, with differences in the pattern and composition of heterochromatin. The second hypothesis seems to be more viable.

In conclusion, these data associated with the previously existing studies for the group, show that, although Curimatidae have a very conservative karyotype macrostructure, the interpopulation variation in NOR locations and distribution of heterochromatin are caused by important mechanisms of chromosome alterations, such as translocations and/or inversions, leading to the evolution and diversification of this group of fish.
